# Propofol alleviates hypoxia-induced nerve injury in PC-12 cells by up-regulation of microRNA-153

**DOI:** 10.1186/s12871-018-0660-z

**Published:** 2018-12-22

**Authors:** Mingwei He, Haiyan Sun, Jinlei Pang, Xiangfei Guo, Yansong Huo, Xianhong Wu, Yaguang Liu, Jun Ma

**Affiliations:** Department of Anesthesiology, Beijing Anzhen Hospital, Capital Medical University, No.2, Anzhen Road, Chaoyang District, Beijing, 100029 China

**Keywords:** Hypoxia injury, PC-12 cells, Propofol, miR-153, BTG3, mTOR/AMPK

## Abstract

**Background:**

Although the neuroprotective role of propofol has been identified recently, the regulatory mechanism associated with microRNAs (miRNAs/miRs) in neuronal cells remains to be poorly understood. We aimed to explore the regulatory mechanism of propofol in hypoxia-injured rat pheochromocytoma (PC-12) cells.

**Methods:**

PC-12 cells were exposed to hypoxia, and cell viability and apoptosis were assessed by CCK-8 assay and flow cytometry assay/Western blot analysis, respectively. Effects of propofol on hypoxia-injured cells were measured, and the expression of miR-153 was determined by stem-loop RT-PCR. After that, whether propofol affected PC-12 cells under hypoxia via miR-153 was verified, and the downstream protein of miR-153 as well as the involved signaling cascade was finally explored.

**Results:**

Hypoxia-induced decrease of cell viability and increase of apoptosis were attenuated by propofol. Then, we found hypoxia exposure up-regulated miR-153 expression, and the level of miR-153 was further elevated by propofol in hypoxia-injured PC-12 cells. Following experiments showed miR-153 inhibition reversed the effects of propofol on hypoxia-treated PC-12 cells. Afterwards, we found BTG3 expression was negatively regulated by miR-153 expression, and BTG3 overexpression inhibited the mTOR pathway and AMPK activation. Besides, hypoxia inhibited the mTOR pathway and AMPK, and these inhibitory effects could be attenuated by propofol.

**Conclusion:**

Propofol protected hypoxia-injured PC-12 cells through miR-153-mediataed down-regulation of BTG3. BTG3 could inhibit the mTOR pathway and AMPK activation.

## Background

The brain is critically dependent upon a continuous blood-borne supply of oxygen, since the normal brain metabolism exerts a high demand of oxygen whereas a limited intrinsic stores of high-energy phosphate compounds [1]. Deprivation of enough oxygen in the brain, induced by a clot within an artery, may ultimately lead to stroke [2]. Reported as the fourth leading cause of death in the United States, stroke is a devastating neural event which causes serious long-term disability [3, 4]. Although the recombinant tissue plasminogen activator (rtPA) has been applied to improve morbidity and mortality of stroke, the therapeutic window is limited [5]. Innovative neuroprotective strategies for stroke are pressingly needed to improve the prognosis of stroke.

Propofol is a widely used intravenous anesthetic agent with rapid onset and short recovery time [6]. It is an agonist on Gamma-Aminobutyric acid (GABA) receptors, and the tumor suppressive role of propofol as well as the carcinogenesis of propofol has been well documented [7–9]. Recently, accumulating evidence has reported the protective roles of propofol against hypoxia in diverse cell types, including neuronal cells [10, 11]. For instance, the neuroprotective role of propofol had been verified in hypoxic hippocampal slice [12]. The CoCl_2_-induced hypoxia injury in mouse hippocampal HT22 cells was attenuated by porpofol through the PP2A/CAMKIIα/nNOS pathway [13]. A previous study also proposed that propofol provided neuroprotection to injured neuronal cells through inhibiting the Toll-like receptor 4 and nuclear factor κB signaling pathways [14]. Although the regulatory mechanisms of propofol in neuronal cells have been studied previously, innovative regulatory pathways are waiting to be identified in order to accelerate the application of propofol in the area of neuroprotection.

MicroRNAs (miRNAs/miRs) are small regulatory RNAs which lack protein coding capacity [15]. Despite of the critical regulatory role of miRNAs in cancer progression, the functional roles of miRNAs such as miR-210 and miR-147 in hypoxia-injured neuronal cells have been widely reported [16, 17]. Although it has been proven that propofol might affect hypoxia-injured cells through regulating miRNAs [18], the regulatory mechanism associated with miRNAs in neuronal cells remains to be poorly understood. MiR-153 is an important tumor regulatory miRNA, acting as either a tumor suppressor or an oncogene in diverse cancer types [19, 20]. Recently, a literature by Liang et al. stated that miR-153 level was up-regulated after hypoxia treatment in breast cancer cells [21]. Kim et al. found miR-153 expression was significantly up-regulated after propofol treatment in adipocyte stem cells, showing approximately 4-fold difference from untreated cells [22]. Additionally, the neuroprotective role of miR-153 has been proven by a previous literature [23]. Those observations described above suggesting that miR-153 might be a downstream miRNA of propofol.

In this study, we constructed in vitro hypoxia cell model using PC-12 cells, and then verified the neuroprotective role of propofol. Furthermore, the possible downstream miRNA of propofol as well as the regulatory axis was also investigated to explain the regulatory mechanism of propofol. Besides, the alteration of involved signaling cascades was also studied.

## Methods

### Cell culture and treatments

The PC-12 cells (ATCC® CRL-1721™) were obtained from American Type Culture Collection (ATCC; Manassas, VA, USA). Cells were maintained in RPMI 1640 medium (Hyclone, Logan, UT, USA) containing 10% heat-inactivated horse serum (Hyclone) and 5% fetal bovine serum (Hyclone). For incubation under normoxia, cells were grown in a humidified incubator at 37 °C with 5% CO_2_ and 95% air. For incubation under hypoxia, cells were subjected into a humidified anaerobic chamber containing 94% N_2_, 5% CO_2_, and 1% O_2_ for 48 h. Propofol (Sigma-Aldrich, St. Louis, MO, USA) was primarily dissolved in dimethyl sulfoxide (DMSO) shortly before use, and it was diluted into RPMI 1640 medium to yield a final propofol concentration of 10 μg/mL. Propofol (10 μg/mL) was applied to stimulate PC-12 cells for 48 h. The final concentration of DMSO in the control group and propofol group was 0.1%. The culture medium was refreshed every other day.

### Transient transfection with miRNAs

Scramble miRNAs, miR-153 mimic, miR-153 inhibitor, and negative control of miR-153 inhibitor (NC) were purchased from GenePharma Co. (Shanghai, China). Each miRNA was transfected into PC-12 cells using Lipofectamine 3000 reagent (Invitrogen, Carlsbad, CA, USA) following the manufacturer’s protocol.

### Generation of stably transfected cells

Short-hairpin RNA directed against BTG3 or a non-targeting sequence was sub-cloned into pGPU6 plasmid (GenePharma), and the recombined plasmids were referred to as sh-BTG3 and sh-NC, respectively. Full-length BTG3 sequence was amplified and inserted into pcDNA3.1 plasmid (GenePharma) to construct pc-BTG3. Recombined plasmids as well as pcDNA3.1 were transfected into PC-12 cells using the lipofectamine 3000 reagent (Invitrogen) according to the manufacturer’s instructions. Stable transfection was selected after incubation in culture medium containing 0.5 mg/ml G418 (Sigma-Aldrich) for approximately 4 weeks.

### Cell viability assay

Cells, seeded in 96-well plates at a density of 5 × 10^3^ cells/well, were incubated at 37 °C overnight. Then, after treatments, 10 μL of Cell Counting Kit-8 (CCK-8) solution, provided by the Dojindo Molecular Technologies (Gaithersburg, MD, USA) was added into the culture medium. Cells were incubated at 37 °C for additional 1 h, and the absorbance at 450 nm was measured using a Microplate Reader (Bio-Rad, Hercules, CA, USA).

### Apoptosis assay

After treatments, cells were trypsinized (0.25% trypsin-EDTA) and collected. Then, cells were washed in cold phosphate-buffered saline (PBS), resuspended in binding buffer provide by the FITC Annexin V/Dead Cell Apoptosis Kit (Invitrogen), and were stained with FITC Annexin V and propidium iodide (PI) according to the instructions of the kit. Stained cells were identified and quantified using a FACS can (Beckman Coulter, Fullerton, CA, USA). Data were analyzed by using FlowJo software (Tree Star, San Carlos, CA, USA).

### miRNA detection by stem-loop reverse transcription (RT)-PCR

After treatments, total RNA was extracted from treated cells using the RNAeasy Mini Kit (Qiagen Sciences, Germantown, MD, USA). cDNAs specific to miRNAs were synthesized using the Taqman MicroRNA Reverse Transcription Kit (Applied Biosystems, Foster City, CA, USA) in line with the suggested procedure. The thermocycling program was shown as follows: 30 min at 16 °C, 30 min at 42 °C and 5 min at 85 °C. Afterwards, real-time PCR was performed with the help of the Taqman Universal Master Mix II (Applied Biosystems) according to the manufacturer’s protocol, and cDNA (50 ng) was acted as the template. The thermal cycling conditions were 1 cycle at 95 °C for 10 min, followed by 40 cycles of 95 °C for 15 s and 60 °C for 1 min. Relative expression of miR-153 was calculated on the basis of the 2^-ΔΔCt^ method [24], and U6 was acted as the housekeeping gene.

### Western blot analysis

After treatments, treated cells were suspended in RIPA buffer (Beyotime, Shanghai, China), and the proteins in the supernatants of the whole lysates were quantified using the BCA Assay Kit (Beyotime). Protein samples (40 μg/lane) were separated by SDS-PAGE, followed by blotting onto the polyvinylidene difluoride (PVDF) membranes. After blocking in 5% non-fat milk at room temperature for 1 h, membranes were incubated with primary antibodies and HRP-conjugated secondary antibodies (goat anti-rabbit, ab97051; goat anti-mouse, ab205719; both Abcam, Cambridge, UK), successively. Primary antibodies included antibodies against cleaved caspase-3 (ab49822), pro caspase-3 (ab90437), B cell lymphoma-2 (Bcl-2; ab196495), Bcl-2-associated X protein (Bax; ab182733), mechanistic target of rapamycin (mTOR, ab2732), phospho (p)-mTOR (ab137133), β-actin (ab8229, all Abcam), B-cell translocation gene 3 (BTG3; TA323849, OriGene Technologies, Inc., Rockville, MD, USA), caspase-9 (#9508), p70S6K (#9202), p-p70S6K (#9205), AMP-activated protein kinase (AMPK; #5832) or p-AMPK (#50081, all Cell Signaling Technology, Beverly, MA, USA). Membranes were then washed by Tris-buffered saline containing 0.1% Tween-20 (TBST). Proteins in the PVDF membranes were visualized by using an enhanced chemiluminescence kit (GE Healthcare Bio-Sciences, Pittsburgh, PA, USA). The intensity of the bands was determined by ImageJ software (National Institutes of Health, Bethesda, MA, USA).

### Statistical analysis

Experiments were performed in triplicate with three repeats. The results were presented as the mean ± standard deviation (SD). Statistical analysis was performed using Graphpad Prism 5 software (GraphPad, San Diego, CA, USA). The *P*-values were calculated using unpaired two-tailed *t*-test, one-way or two-way analysis of variance (ANOVA). A *P* < 0.05 was considered as a significant difference.

## Results

### Hypoxia induced PC-12 cell apoptosis

**Fig. 1:**
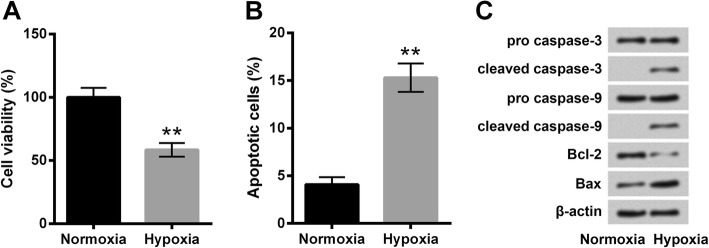
Hypoxia induced PC-12 cell apoptosis. **a** Cell viability by CCK-8. **b** Percentage of apoptotic cells by flow cytometry assay. **c** Expression of apoptosis-related proteins by Western blot analysis. PC-12 cells were incubated under normoxia or hypoxia for 48 h. Data was presented as the mean ± standard deviation (SD) of three independent experiments. **, *P* < 0.01

After hypoxia treatments, cell viability and apoptosis of PC-12 cells were evaluated. Compared with cells under normoxia, cell viability was markedly reduced by hypoxia exposure (*P* < 0.01, Fig. 1a). Percentage of apoptotic cells in the Hypoxia group was notably higher than that in the Normoxia group (*P* < 0.01, Fig. 1b). Western blot results in Fig. 1c showed expression of pro-apoptotic cleaved caspase-3, cleaved caspase-9 and Bax was observably up-regulated after hypoxia exposure, whereas expression of anti-apoptotic Bcl-2 was down-regulated after hypoxia exposure. Results proposed that in vitro hypoxia model was constructed successfully in PC-12 cells.

### Propofol alleviated hypoxia-induced apoptosis in PC-12 cells

**Fig. 2:**
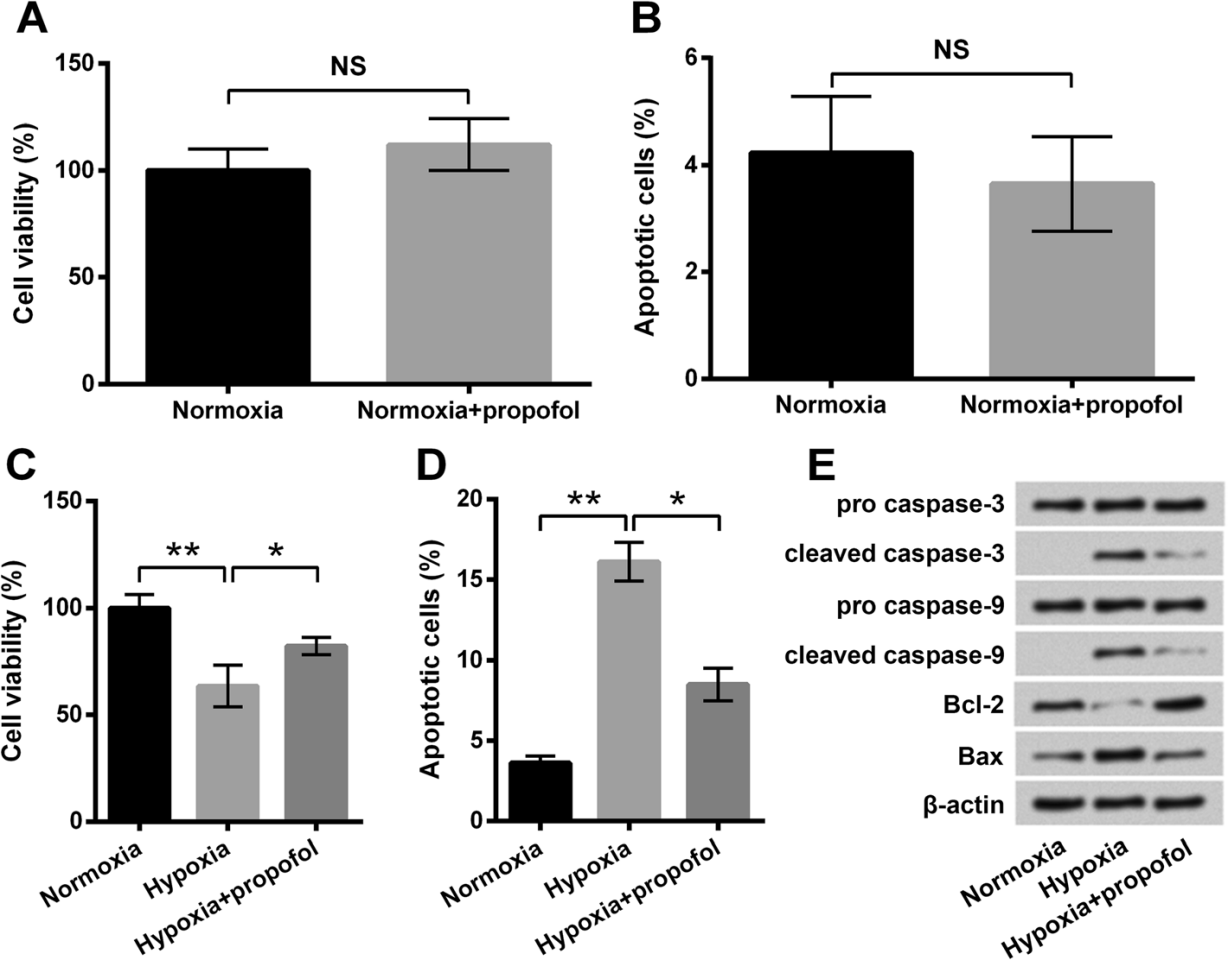
Hypoxia-induced PC-12 cell apoptosis was alleviated by propofol. **a** Cell viability by CCK-8. **b** Percentage of apoptotic cells by flow cytometry assay. **c** Cell viability by CCK-8. **d** Percentage of apoptotic cells by flow cytometry assay. **e** Expression of apoptosis-related proteins by Western blot analysis. PC-12 cells were incubated under normoxia or hypoxia with or without 10 μg/mL propofol for 48 h. Data was presented as the mean ± standard deviation (SD) of three independent experiments. NS, *P* > 0.05; *, *P* < 0.05; **, *P* < 0.01

PC-12 cells under normoxia were treated with propofol, and cell viability and apoptosis were measured. As shown in Fig. 2a, difference of viability between propofol-treated cells and untreated cells was non-significant (*P* > 0.05). Propofol also showed a non-significant effect on apoptosis in cells under normoxia (*P* > 0.05, Fig. 2b). After that, cells were exposed to hypoxia with or without propofol, and then cell viability and apoptosis were measured. Compared with the Hypoxia group, cell viability was markedly enhanced (*P* < 0.05, Fig. 2c) and apoptosis was notably decreased (*P* < 0.05, Fig. 2d) by propofol. In the meantime, alteration of apoptosis-related proteins was also attenuated by propofol (Fig. 2e). Therefore, we concluded that propofol could attenuate hypoxia-induced apoptosis in PC-12 cells.

### Propofol up-regulated miR-153 expression in PC-12 cells

**Fig. 3:**
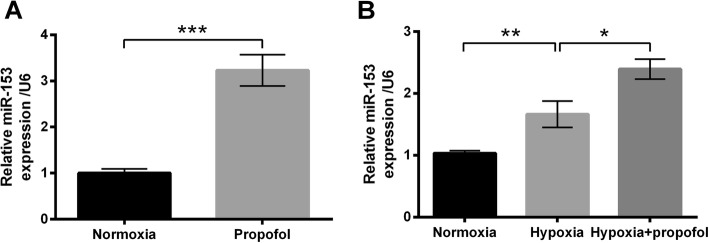
Propofol up-regulated miR-153 expression in PC-12 cells. **a** Expression of miR-153 by stem-loop RT-PCR. Cells were incubated under hypoxia with or without propofol for 48 h. Cells in the Normoxia group were incubated under normoxia for 48 h. PC-12 cells were incubated under normoxia with or without 10 μg/mL propofol for 48 h. **b** Expression of miR-153 by stem-loop RT-PCR. PC-12 cells were incubated under normoxia or hypoxia with or without 10 μg/mL propofol for 48 h. Data was presented as the mean ± standard deviation (SD) of three independent experiments. *, *P* < 0.05; **, *P* < 0.01; ***, *P* < 0.001

Cells under normoxia were stimulated with propofol, and results in Fig. 3a showed propofol dramatically up-regulated miR-153 expression compared with the Normoxia group (*P* < 0.001). After exposure to hypoxia with or without propofol, the expression of miR-153 in PC-12 cells was also determined. As evidenced in Fig. 3b, we found miR-153 level was significantly elevated by hypoxia treatments (*P* < 0.01), and the elevation was further increased by propofol (*P* < 0.05). Results suggested that miR-153 might be involved in the regulatory effects of propofol on hypoxia-treated PC-12 cells.

### Propofol protected PC-12 cells against hypoxia through up-regulating miR-153

**Fig. 4:**
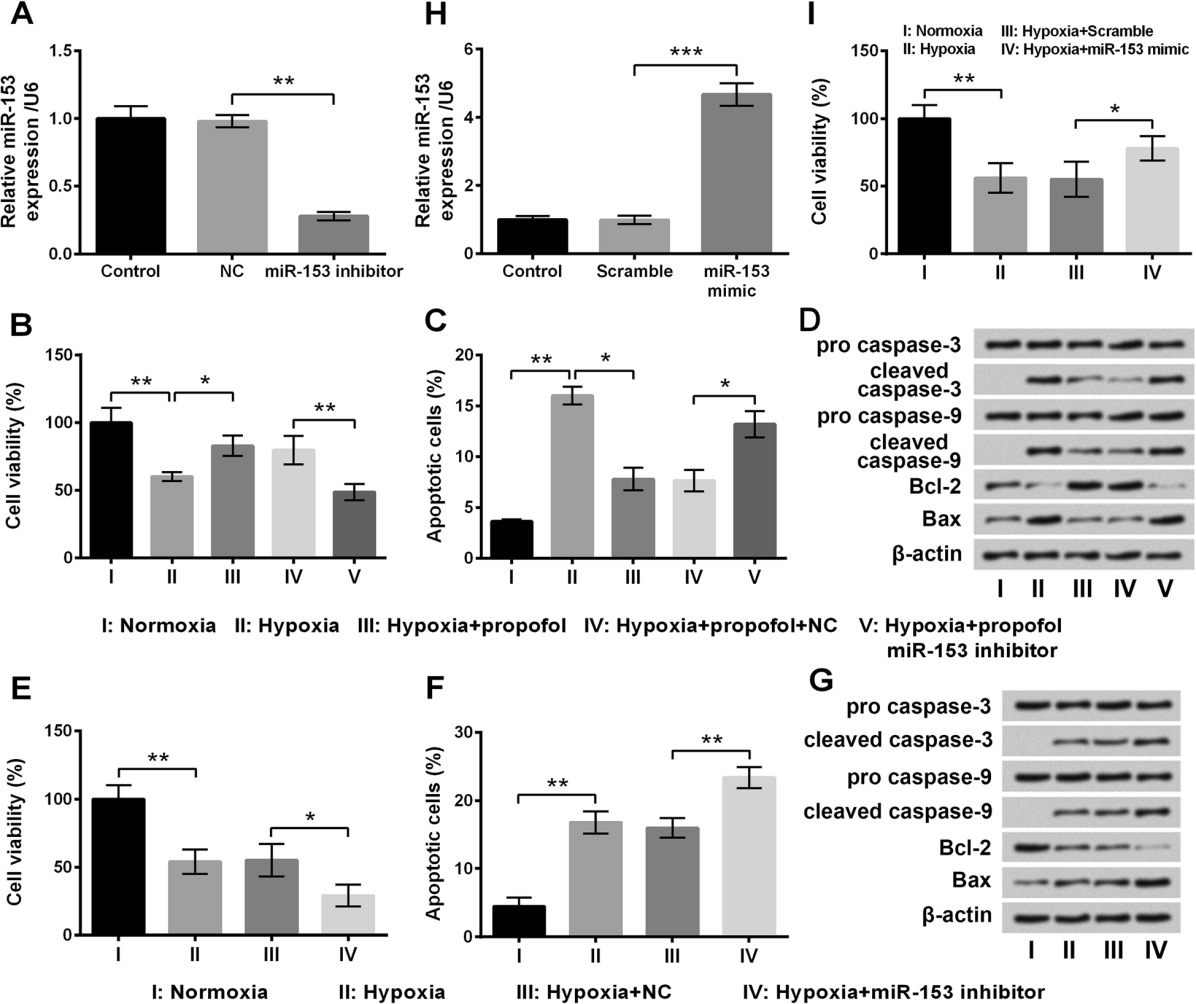
Effects of propofol on hypoxia-injured PC-12 cells were reversed by miR-153 inhibition. **a** Expression of miR-153 by stem-loop RT-PCR. Cells were divided into five groups, including Normoxia, Hypoxia, Hypoxia + propofol, Hypoxia + propofol + NC, and Hypoxia + propofol + miR-153 inhibitor. The duration of treatments was 48 h. **b** Cell viability by CCK-8. **c** Percentage of apoptotic cells by flow cytometry assay. **d** Expression of apoptosis-related proteins by Western blot analysis. Cells were divided into four groups, including Normoxia, Hypoxia, Hypoxia + NC, and Hypoxia + miR-153 inhibitor. The duration of treatments was 48 h. **e** Cell viability by CCK-8. **f** Percentage of apoptotic cells by flow cytometry assay. **g** Expression of apoptosis-related proteins by Western blot analysis. Cells were transfected with scramble miRNAs or miR-153 mimic. **h** Expression of miR-153 by stem-loop RT-PCR. **i** Cell viability by CCK-8 assay. Cells were divided into four groups, including Normoxia, Hypoxia, Hypoxia + Scramble, and Hypoxia + miR-153 mimic. The duration of treatments was 48 h. Data was presented as the mean ± standard deviation (SD) of three independent experiments. *, *P* < 0.05; **, *P* < 0.01; ***, *P* < 0.001

After transfection with miRNAs, miR-153 level in cells transfected with miR-153 inhibitor was markedly lower than that in cells transfected with NC (*P* < 0.01, Fig. 4a), indicating that cell transfection could down-regulate miR-153 expression successfully. Then, transfected or untransfected cells were exposed to hypoxia with or without propofol, and then cell viability and apoptosis were analyzed. Results showed effects of propofol on PC-12 cells under hypoxia could be significantly reversed by miR-153 inhibition, as evidenced by decreased cell viability (*P* < 0.01, Fig. 4b), increased apoptotic cells (*P* < 0.05, Fig. 4c), up-regulation of cleaved caspase-3, cleaved caspase-9 and Bax, and down-regulation of Bcl-2 (Fig. 4d). Moreover, the effects of miR-153 on hypoxia-treated cells were explored to verify whether alteration of miR-153 could effects hypoxia-treated cells. Results showed effects of hypoxia were augmented by miR-153 inhibition, as miR-153 inhibition significantly decreased cell viability (*P* < 0.05, Fig. 4e) while markedly increased apoptotic cells (*P* < 0.01, Fig. 4f), as compared to the Hypoxia + NC group. As shown in Fig. 4h, miR-153 level in cells transfected with miR-153 mimic was remarkably higher than that in cells transfected with Scramble miRNA (*P* < 0.001), indicating that cell transfection could up-regulate miR-153 expression successfully. Results in Fig. 4i showed miR-153 overexpression could effectively enhanced cell viability compared with the Hypoxia + Scramble group (*P* < 0.05). Results collectively suggested that propofol might affect hypoxia-injured PC-12 cells through up-regulating miR-153 expression.

### BTG3 expression was negatively correlated with miR-153 expression

**Fig. 5:**
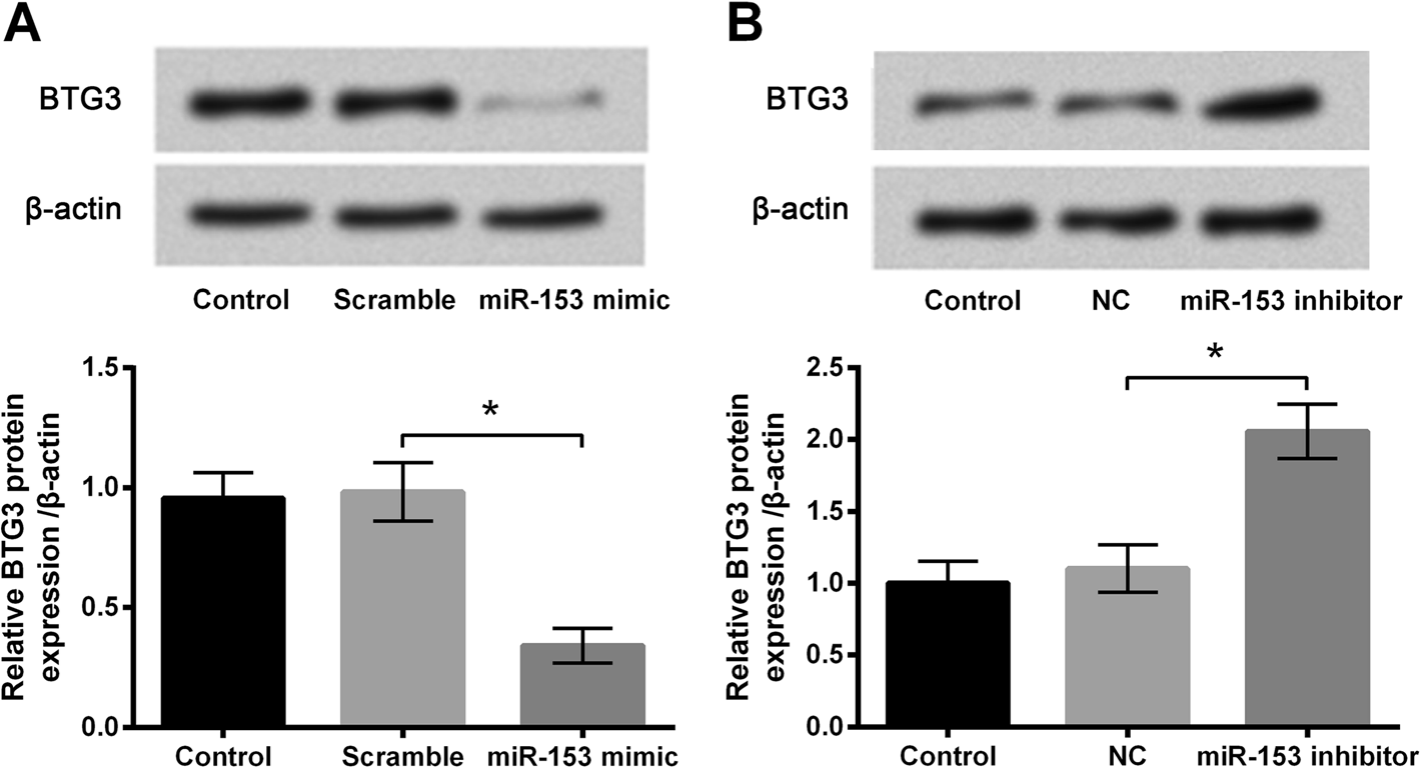
BTG3 was negatively regulated by miR-153 expression in PC-12 cells. **a** PC-12 cells were transfected with scramble miRNAs or miR-153 mimic. **b** PC-12 cells were transfected with miR-153 inhibitor or its negative control (NC). Protein expression of BTG3 was measured by Western blot analysis. Untransfected cells were acted as control Data was presented as the mean ± standard deviation (SD) of three independent experiments. *, *P* < 0.05

The protein expression of BTG3 in PC-12 cells abnormally expressing miR-153 was assessed. In Fig. 5a, BTG3 protein expression level in cells overexpressing miR-153 was significantly lower than that in the Scramble group (*P* < 0.05). In the meantime, BTG3 protein expression level in cells silencing miR-153 was significantly higher than that in the NC group (*P* < 0.05, Fig. 5b). Results indicated that BTG3 expression was negatively correlated with miR-153 expression in PC-12 cells.

### BTG3 overexpression inactivated the mTOR pathway and AMPK activation in PC-12 cells

**Fig. 6:**
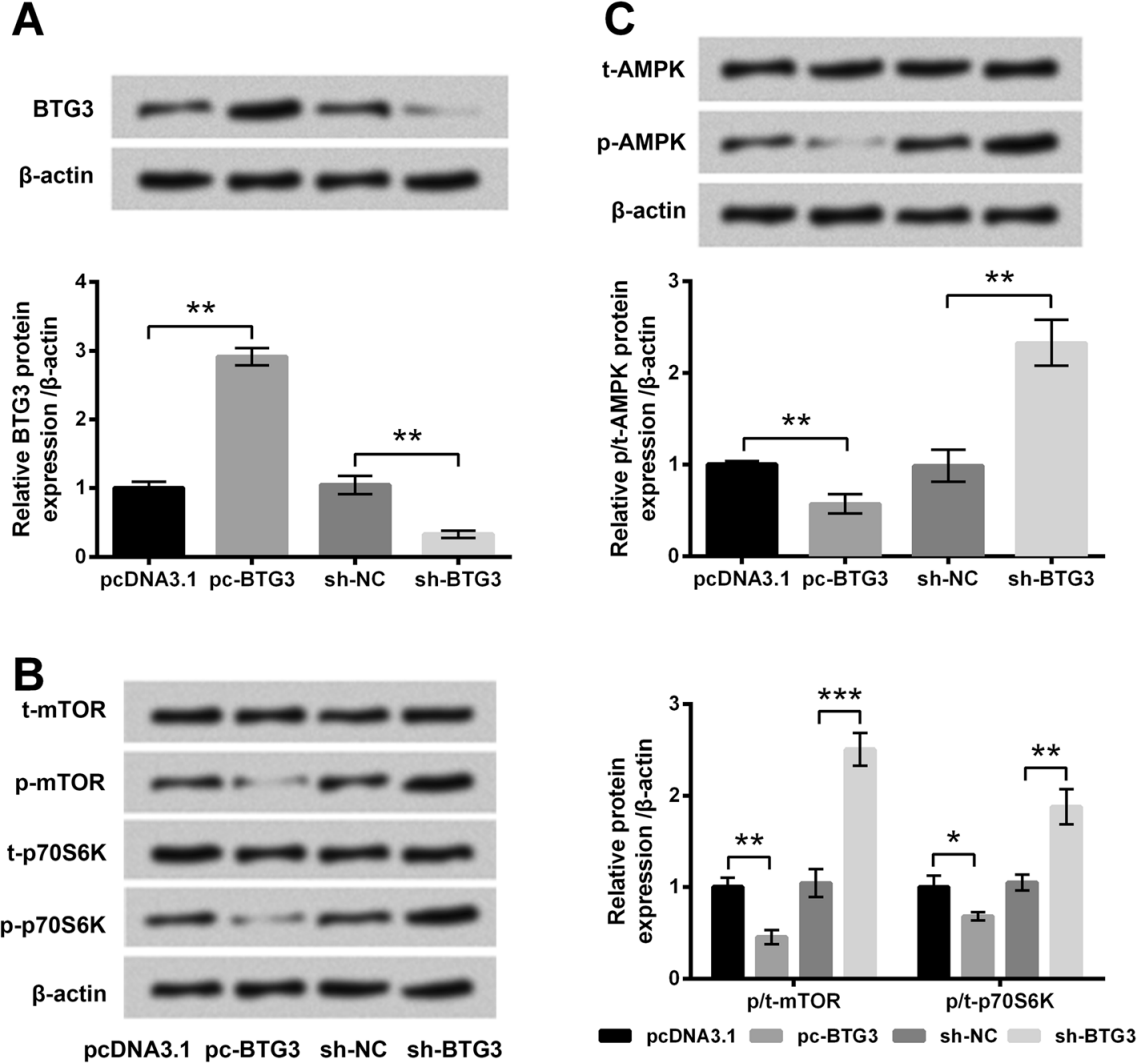
BTG3 overexpression inhibited the mTOR pathway and AMPK activation in PC-12 cells. **a** Protein expression of BTG3 was assessed by Western blot analysis. Phosphorylated levels of key kinases in the mTOR pathway (**b**) and AMPK (**c**) were assessed by Western blot analysis. PC-12 cells were transfected with pcDNA3.1, pc-BTG3, sh-NC or sh-BTG3. Data was presented as the mean ± standard deviation (SD) of three independent experiments. *, *P* < 0.05; **, *P* < 0.01; ***, *P* < 0.001

The effects of BTG3 on activation of the mTOR pathway and AMPK were analyzed. In stably transfected cells, protein expression of BTG3 was testified. As shown in Fig. 6a, BTG3 protein expression level in cells transfected with pc-BTG3 was dramatically higher than that in cells transfected with pcDNA3.1 (*P* < 0.01). Meanwhile, BTG3 protein expression level in cells transfected with sh-BTG3 was significantly lower than that in cells transfected with sh-NC (*P* < 0.01). Results proved that BTG3 expression could be overexpressed or silenced after stable transfection. Then, phosphorylation of AMPK and key kinases in the mTOR pathway was analyzed. Phosphorylated levels of mTOR, p70S6K and AMPK were markedly reduced by BTG3 overexpression (*P* < 0.05 or *P* < 0.01, Fig. 6b, c) while were dramatically enhanced by BTG3 silence (*P* < 0.01 or *P* < 0.001). Results indicated that BTG3 overexpression could inhibit the mTOR pathway and AMPK activation in PC-12 cells.

### Hypoxia inactivated the mTOR pathway and AMPK activation, and propofol attenuated these inhibitory effects via regulation of BTG3

**Fig. 7:**
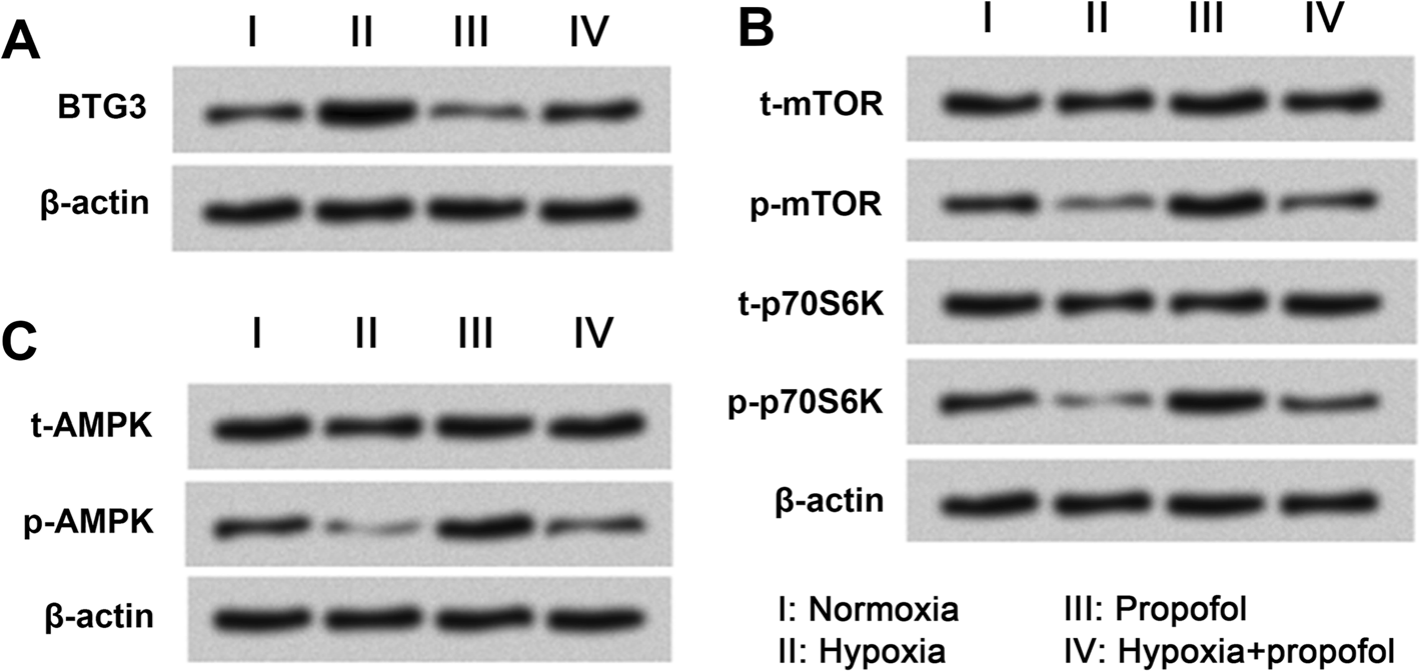
Hypoixa inhibited the mTOR pathway and AMPK activation, and propofol attenuated these inhibitory effects via regulation of BTG3. PC-12 cells were divided into four groups, including Normoxia, Hypoxia, Propofol, and Hypoxia + propofol. **a** Protein expression of BTG3 by Western blot analysis. Phosphorylated levels of key kinases in the mTOR pathway (**b**) and AMPK (**c**) were assessed by Western blot analysis

The effects of hypoxia and/or propofol on BTG3 expression were evaluated. As shown in Fig. 7a, BTG3 expression was up-regulated by hypoxia while was down-regulated by propofol, and the effects of hypoxia could be alleviated by propofol. Moreover, we also found the phosphorylated levels of mTOR, p70S6K and AMPK were reduced by hypoxia while were enhanced by propofol (Fig. 7b, c). In addition, the effects of hypoxia on the mTOR pathway and AMPK activation were attenuated by propofol. Results indicated that hypoxia inactivated the mTOR pathway and AMPK activation, and propofol attenuated these inhibitory effects via regulation of BTG3.

## Discussion

PC-12 cells are widely used as a model system to study the mechanisms involved in neuronal cells under hypoxia. For example, the protective role of silent information regulation 2 homolog 3 (SIRT3) in ischemia-induced neuronal death as well as the underlying mechanisms was studied in PC-12 cells [25]. The neuroprotective role of cerebrolysin in hypoxia-induced neuronal cytotoxicity was also studied in PC-12 cells [26]. In the present study, the in vitro protective role of propofol in neuronal cells was studied in PC-12 cells. Data indicated that hypoxia-induced PC-12 cell injury could be alleviated by propofol treatment. Then, we found miR-153 level was elevated after hypoxia treatment and its expression was further up-regulated in cells treated with hypoxia and propofol. Additional experiments revealed that propofol functioned through up-regulating miR-153 in hypoxia-exposed cells. Afterwards, we found miR-153 expression negatively regulated BTG3 expression, and BTG3 inhibited the mTOR pathway and AMPK activation in PC-12 cells.

In the first step of our study, the effects of propofol on hypoxia-treated cells were measured. Although propofol showed non-significant effects on viability and apoptosis in cells under normoxia, the hypoxia-induced decrease of cell viability as well as increase of apoptosis was mitigated by propofol in PC-12 cells. Apoptosis is a process of programmed cell death, in which several changes were observed, such as alteration of Bcl-2 family proteins, activation of caspases and DNA fragmentation [27]. In our study, hypoxia-induced up-regulation of Bax, cleaved caspase-3 and cleaved caspase-9 as well as down-regulation of Bcl-2 was obviously attenuated by propofol, implying that propofol might alleviate hypoxia-induced PC-12 cell injury through repressing the mitochondrial- and caspase-dependent pathways. The protective role of propofol against hypoxia injury in PC-12 cells was consistent with that in other cell types. For example, cell death induced by hypoxia in alveolar epithelial type II cells of rats could be reduced by propofol [10]. hypoxia/reoxygenation-induced apoptosis of human brain vascular smooth muscle cells was attenuated by propofol [11].

Next, we explored whether there was a correlation between propofol and miR-153 in hypoxia-injured PC-12 cells. Intriguingly, our results found miR-153 level was up-regulated by hypoxia, and its level was further up-regulated by propofol in PC-12 cells under normoxia or hypoxia. An evidence suggested that the increased expression of miR-153 occurred in cells in response to external stimulus, for instance, neurotoxin [23]. Similarly, although it’s still unexplored why hypoxia could up-regulate the expression of miR-125, we considered that the expression of miR-125 was enhanced against hypoxia-induced injury. However, the increased miR-125 could not thoroughly repress the expression of hypoxia-inducible factor (HIF)-1α, which was inhibited by the overexpression of miR-153 for inhibiting apoptosis and enhancing proliferation [28, 29]. By adequately enhancing the expression of miR-153, propofol exerted a cytoprotection against hypoxia-induced injury. Moreover, neuroprotective effects of propofol on hypoxia-injured PC-12 cells could be abrogated by miR-153 knockdown, indicating that up-regulated miR-153 might be a possible reason for the neuroprotective role of propofol. To support the conclusion, we also proved that hypoxia injury was heightened by miR-153 inhibition while was attenuated by miR-153 overexpression. A previous study has proposed the negative regulation between miR-153 and hypoxia-inducible factor (HIF)-1α [29]. However, the effects of HIF-1α on hypoxia-injury are controversial. Liu et al. have shown that down-regulation of HIF-1α increases apoptosis of hypoxic cardiomyocytes [30]. Chen et al. have reported that HIF-1α knockdown alleviates neonatal brain damage after hypoxic injury. Therefore, there might be another downstream factor of miR-153 in the regulation of propofol.

*BTG3* belonging to anti-proliferative BTG gene family has been reported as a tumor suppressor gene [31]. A previous study has illustrated that BTG3 overexpression showed higher expression of Bax, caspase-3 and caspase-9 [32]. Considering the observable effects of propofol on those proteins associated with apoptosis, we speculated that BTG3 might participate in the regulatory mechanism of propofol. Results in our study found BTG3 expression was negatively regulated by miR-153. A previous study has proved that BTG3 is a direct target of p53 [33]. Krüppel-like factor 5 (KLF5) is a target of miR-153 [34] which can interact with p53 [35]. Those observations described above might provide a rational explanation for the negative correlation between miR-153 and BTG3. The mTOR pathway governing cellular response to hypoxia plays critical role in regulating cell death under environmental stress [36]. AMPK is a stress-responsive enzyme involved in cell adaptation to an energy crisis [37]. We further found that BTG3 overexpression could inhibited the mTOR pathway and AMPK activation, and BTG3 silence showed the opposite effects.

In the end, we also studied the effects of hypoxia and/or propofol on BTG3 expression and phosphorylation of mTOR, p70S6K and AMPK, in order to verify the regulatory axis of propofol-miR-153-BTG3. Western blot results showed hypoxia up-regulated BTG3 expression while propofol down-regulated BTG3 expression, and the hypoxia-induced BTG3 abundance was decreased by propofol. The effects of hypoxia and/or propofol on the mTOR pathway and AMPK activation could substantiate the effects of BTG3 on the mTOR pathway and AMPK activation.

## Conclusions

To summarize, we verified the protective role of propofol in hypoxia-exposed PC-12 cells and found propofol might affect PC-12 cells under hypoxia through miR-153-mediated down-regulation of BTG3. BTG3 expression overexpression inhibited the mTOR pathway and AMPK activation. This study provided basis for the study of propofol function, assisting in discovery of innovative strategies for clinical neuroprotection.
